# A nationwide study on concordance with multimodal treatment guidelines in bipolar disorder

**DOI:** 10.1186/s40345-018-0130-z

**Published:** 2018-10-20

**Authors:** Joannes W. Renes, Eline J. Regeer, Adriaan W. Hoogendoorn, Willem A. Nolen, Ralph W. Kupka

**Affiliations:** 1 Altrecht Institute for Mental Health Care, Utrecht, Nieuwe Houtenseweg 12, 3524 SH Utrecht, The Netherlands; 2 Amsterdam UMC, Vrije Universiteit Amsterdam, Psychiatry, Amsterdam Public Health, De Boelelaan 1117, 1081 HV Amsterdam, The Netherlands; 3 Department of Psychiatry, University Medical Center Groningen, University of Groningen, Hanzeplein 1, 9713 GZ Groningen, The Netherlands

**Keywords:** Bipolar, Guidelines, Concordance

## Abstract

**Background:**

Most previous studies on concordance with treatment guidelines for bipolar disorder focused on pharmacotherapy. Few studies have included other treatment modalities.

**Aims:**

To study concordance with the Dutch guideline of various treatment modalities in outpatient treatment settings for patients with bipolar disorder and to identity factors associated with concordance.

**Methods:**

A nationwide non-interventional study using psychiatrists’ and patients’ surveys.

**Results:**

839 patients with bipolar or schizoaffective disorder bipolar type were included. Concordance with the guideline was highest for participation of a psychiatrist in the treatment (98%) and for maintenance pharmacotherapy (96%), but lower for supportive treatment (73.5%), use of an emergency plan (70.6%), psychotherapy (52.2%), group psychoeducation (47.2%), and mood monitoring (47%). Presence of a written treatment plan, a more specialized treatment setting, more years of education, and diagnosis of bipolar I disorder versus bipolar II, bipolar NOS, or schizoaffective disorder were significantly associated with better concordance.

**Conclusion:**

In contrast to pharmacotherapy, psychosocial treatments are only implemented to a limited extend in everyday clinical practice in bipolar disorder. More effort is needed to implement non-pharmacological guideline recommendations for bipolar disorder.

## Background

To improve the quality of care, several guidelines for the treatment of bipolar disorder (BD) have been published in the past two decades, including in the Netherlands (Kupka et al. 2015; Nolen et al. 2008). Studies on the naturalistic treatment of BD show that concordance with these guidelines varies considerably from less than 50% (Altinbas et al. 2011; Baek et al. 2014; Busch et al. 2007; Lim et al. 2001), 50–70% (Arvilommi et al. 2007; Bauer et al. 2009; Farrelly et al. 2006; Freeland et al. 2015; Huang et al. 2014; Marcus et al. 1999; Simon et al. 2004; Smith et al. 2008; Wang et al. 2014a, 2015), or up to 90% (Altinbas et al. 2011; Farrelly et al. 2006; Kilbourne et al. 2005; Paterniti and Bisserbe 2013; Unutzer et al. 2000; Walpoth-Niederwanger et al. 2012; Wang et al. 2014b) on the primary outcome measure of concordance. These concordance rates are difficult to compare due to differences in study design, treatment settings, and in what phase of the illness concordance was studied. Most studies focused on pharmacotherapy only (including monitoring of plasma levels), and were retrospective in design. Few studies have included other treatment modalities, such as psychoeducation or psychotherapy, or visits with health care providers (Busch et al. 2007; Farrelly et al. 2006; Kilbourne et al. 2005; Unutzer et al. 2000).

In these naturalistic studies on concordance with treatment guidelines, factors that have been found to be of influence are type of mood episode (Baek et al. 2014; Farrelly et al. 2006; Huang et al. 2014; Paterniti and Bisserbe 2013), psychotic features (Altinbas et al. 2011; Lim et al. 2001), bipolar disorder subtype (Simon et al. 2004), age at onset (Dennehy et al. 2007), rapid cycling (Arvilommi et al. 2007), treatment setting (Arvilommi et al. 2007; Busch et al. 2007), race (Kilbourne et al. 2005), and higher medical complexity in elderly patients (Huang et al. 2014).

In 2008 a revised guideline on the treatment of bipolar disorder in the Netherlands was published (Nolen et al. 2008). In this paper we present the outcomes of a nationwide naturalistic prospective study on treatment practice and concordance with this Dutch guideline in various treatment settings for patients with BD or schizoaffective disorder, bipolar type (SZA). We hypothesized that the guideline would be better implemented in centers specialized in the treatment of mood disorders, and in patients with bipolar I disorder (BD I) versus those with bipolar II disorder (BD II), bipolar disorder NOS (BD NOS), or SZA. Since BD I is more clearly defined by the lifetime occurrence of full manic episodes, this diagnosis will represent a more homogeneous group of patients, for which providers probably better recognize treatment recommendations in the guideline. Moreover, guidelines often take BD I as their main focus. We further examined the relationship of demographic, illness, and treatment variables with concordance with the guideline.

## Methods

The Treatment of Bipolar Disorder in the Netherlands study (TBDN) is a nationwide, multicenter, non-intervention study on concordance with guideline recommendations for the long-term treatment of BD and SZA in mental health outpatient treatment settings (Renes et al. 2014). The study was performed between December 2009 and June 2014.

### Selection of psychiatrists and patients

Between December 2009 and February 2010 all psychiatrists registered as member of the Dutch Psychiatric Association received a short survey about their treatment setting and whether they would be willing to participate in this study. All psychiatrists who indicated that they were treating adult patients with BD or SZA in an outpatient setting and were interested in participating in the study, received a questionnaire about their treatment setting, organization of care, and the number of patients currently in treatment for BD or SZA. Furthermore, they were asked to send a letter to all these patients inviting them to participate in the study.

All patients who returned an informed consent form were sent two questionnaires: one for themselves and one for a spouse, relative or significant other. The patients’ questionnaire concerned care they had received in the previous 12 months or earlier for some treatment modalities, lifetime illness characteristics, clinical outcome, quality of life and functioning, satisfaction with care, and adherence to treatment. For each patient a clinical diagnosis, according to DSM-IV-TR (American Psychiatric Association 2000), was supplied by the treating psychiatrist, including comorbid diagnoses. The study was approved by the Medical Ethical Committee of the University Medical Center Utrecht, the Netherlands, and independently reviewed by the scientific committees of the two main participating research centers, Altrecht Institute for Mental Health Care, Utrecht, the Netherlands, and GGZ inGeest/VU University Medical Center, Amsterdam, the Netherlands. All participating patients gave written informed consent.

### Outcome measures

#### Treatment modalities

Patients were asked to tick the medication they were currently using from a list of maintenance medications (lithium, carbamazepine, valproate, lamotrigine, olanzapine, quetiapine, risperidone, or aripiprazole), and to add any other medication they were currently using for BD, and were asked if, and with what frequency, laboratory tests were part of the treatment with lithium, valproate or carbamazepine. For psychosocial treatments, patients were asked if they ever had participated in a group psychoeducation program, if they had ever received psychotherapy, and if so, whether they had received it in the previous year, if they had received supportive treatment in the previous year, if they had an emergency plan on how to deal with early symptoms of an impending mood episode, and if they regularly monitored their mood by completing prospective LifeCharts according to the NIMH Life-Chart Method (Leverich and Post 1998), which is well-known in the Netherlands.

#### Measurement of concordance with the Dutch guideline

In the Dutch guideline recommendations may differ for patients with specific clinical profiles. For our study we distinguished four clinical profiles. Table 1 indicates which treatment modalities are, and which are not, recommended to be part of the treatment for patients with these profiles.

**Table 1:** Treatment modalities that are recommended by the Dutch guideline for the treatment of BD in patients with differential clinical profiles

	Clinical profiles			
	Currently asymptomatic and no indication for maintenance pharmacotherapy	Currently asymptomatic, with an indication for maintenance pharmacotherapy, and *no* episode in the previous year	Currently asymptomatic, with an indication for maintenance pharmacotherapy, and an episode in the previous year	Currently symptomatic
Treatment modality^a^				
Participation of a psychiatrist^b^	+	+	+	+
Group psychoeducation	+	+	+	+
Emergency plan	+/−^c^	+/−^c^	+	+
Maintenance pharmacotherapy	–	+	+	+
Life charting	−	−	+	+
Supportive treatment^d^	−	−	+	+
Psychotherapy^e^	−	−	−	+

^a^“+” indicates the modality is recommended to be part of the treatment in case of that particular clinical profile, and “−” indicates the modality is not recommended to be part of the treatment

^b^Patients with BD should have at least one visit/year with a psychiatrist or physician, when health care providers other than a psychiatrist/physician are part of the treatment team

^c^For these patients an emergency plan is recommended as optional

^d^At least three visits with a psychiatrist or mental health nurse in the previous year

^e^Any form of psychotherapy in the previous year

Maintenance pharmacotherapy is recommended for patients after three or more mood episodes, and for patients after two episodes if at least one of the episodes was severe, or when the patient has a first degree relative with BD. Furthermore, maintenance pharmacotherapy may be considered: (1) after a single severe manic episode, (2) after a single manic episode of any severity and having a first degree relative with BD, or (3) after two non-severe episodes without a family history of BD. Finally, maintenance pharmacotherapy is not recommended for patients with a single non-severe manic episode in the absence of a first degree relative with BD.

To assess concordance with the Dutch guideline, a composite score ranging from 0 to 100 for the degree of concordance was developed based on the sum of scores for each treatment modality, taking into account an assumed impact factor of each treatment modality on treatment outcome as determined by consensus among the authors (JR, ER, WN, RK), when taking into account the level of scientific evidence of recommendations as described in the guideline. Of notice, WN and RK had been involved in the development of the guideline. The impact factors were rated as follows: pharmacotherapy 40 points, group psychoeducation 20, psychotherapy 20, participation of a psychiatrist 5, having an emergency plan on how to deal with emerging symptoms 5, mood monitoring 5, and supportive treatment 5 points.

If, according to the guideline, a treatment modality was recommended and accordingly applied, points were added to the total score; and similarly if a treatment modality was not recommended and accordingly not applied. If a treatment modality was recommended but not applied, or applied despite not being recommended, no points were added. The latter was based on the assumption that a more intensive treatment is not necessarily beneficial and might even pose a psychological or biological burden on the patient. No points were subtracted in case of non-concordance.

#### Assessment of symptoms and illness characteristics

The Quick Inventory of Depressive Symptomatology (QIDS) (Rush et al. 1996), and the Altman Self-Rating Mania Scale (ASRM) (Altman et al. 1997), were part of the patient questionnaire to measure current severity of mood symptoms. The questionnaire also addressed various lifetime illness characteristics. Because these data were obtained through self-reporting, some data could be missing or conflicting. When data were conflicting, consensus was first reached between the first two authors (JR, ER). The other authors (WN, RK) were consulted when necessarily. Data that remained inconclusive were excluded from analysis.

### Statistical analyses

SPSS 22 was used for statistical analysis. Descriptive statistics were used for demographics and illness characteristics of the sample. Relationships with the total score of concordance were tested using simple and multiple regression analyses.

## Results

### Inclusion

**Fig. 1:**
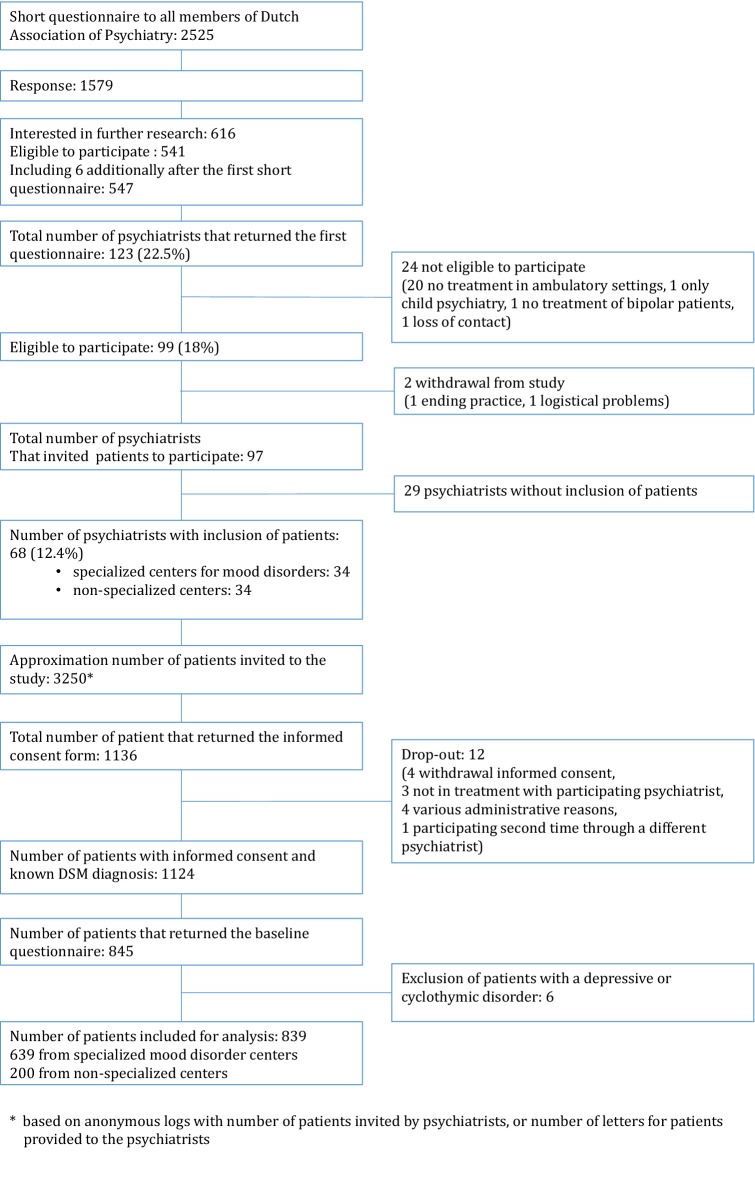
Inclusion of psychiatrists and patients

The inclusion of psychiatrists and patients is presented in Fig. 1.

### Socio-demographic and illness characteristics

Five hundred and fifty-five respondents (66.2%) were women. The average age was 49.5 years (s.d. 11.2). Five hundred and one out of 833 (60.1%) respondents were married or living together. The mean years of education (n = 837) was 16.1 years (s.d. 4.3). Diagnoses were BD I (n = 551; 65.7%), BD II (n = 211; 25.1%), BD NOS (n = 32; 3.8%), and SZA (n = 45; 5.4%). At least one comorbid psychiatric diagnosis was present in 238 respondents (28.4%). The mean duration of illness (n = 712) was 23.8 years (s.d. 12.3). The average age at onset for (hypo)manic symptoms was 30.1 years (s.d. 11.8), and for depressive symptoms 26.1 years (s.d. 11.7). Only nine respondents (1.1%) experienced one single manic episode, 28 (3.3%) had two mood episodes, all other 718 respondents (85.6%) had three or more mood episodes. Data on total lifetime number of mood episodes were inconclusive or missing in 84 respondents (10%). Hospital admission because of a mood episode was reported by 532 (63.4%) respondents. Data on admission were missing in 65 respondents (7.7%). Of the respondents, 283 (33.7%) had a first degree relative with bipolar disorder.

### Treatments

The number of respondents receiving maintenance pharmacotherapy and various forms of psychosocial treatments is reported in Table 2.

**Table 2:** Elements of current treatment as reported by the patients

Treatment modalities^a^	*n*	%
Current use of maintenance medication (n = 836)		
Lithium	590	70.6
Anticonvulsants^b^	281	33.6
Atypical antipsychotics^c^	318	38.0
Conventional antipsychotics	30	3.6
Ever participated in group psychoeducation (n = 836)	394	47.1
Emergency plan (n = 836)	484	57.9
Participation of a psychiatrist (n = 819)	803	98.0
Mood monitoring (n = 835)	229	27.4
Psychotherapy in the previous year (n = 681)	133	15.9
Supportive treatment (n = 809)	749	92.6
Patient reports that a treatment plan has been made (n = 825)		
Yes	502	60.8
No	323	39.2
Patient is involved in decision-making (n = 827)		
Never	52	6.3
Sometimes	166	20.1
Mostly	279	33.7
Always	330	39.9
Significant others have been asked to participate in the treatment (n = 828)		
Yes	677	81.8
No	151	18.2
Patient uses the internet for information on bipolar disorders and treatments (n = 836)		
Yes	357	42.7
No	479	57.3

^a^Sample size may differ among variables, depending on missing data points

^b^Valproate, lamotrigine, carbamazepine

^c^Olanzapine, quetiapine, risperidone, aripiprazole, clozapine

#### Maintenance pharmacotherapy

Lithium, carbamazepine, valproate, lamotrigine or an antipsychotic as maintenance medication was used by 804 (96.1%) respondents. Of the remaining 32 (3.9%) who currently did not use any pharmacotherapy, 15 had BD I, 13 BD II, three BD NOS, and one SZA; all reported at least two previous mood episodes (data missing in one), and eight had been admitted at least once (data missing in two).

Polypharmacy was common. When all medications, excluding benzodiazepines and somatic medications, were taken together, 328 (39.1%) respondents used two drugs, and 117 (13.9%) three or more drugs with a maximum of five. In addition to the medication already listed in the questionnaire, 169 (20.1%) respondents reported the use of an antidepressant (14.2% BD I; 35.5% BD II; 25.0% BD NOS; 17.8% SZA). In 15 of these 169 respondents (BD I n = 4; BD II = 10; BD NOS = 1) the antidepressant was not combined with lithium, an anticonvulsant or an antipsychotic. Of the respondents using either lithium, valproate or carbamazepine (n = 711), almost all (n = 702) reported an adequate frequency of laboratory testing.

#### Psychotherapy

Of the 133 respondents that received psychotherapy in the previous year, 77 (57.8%) reported that the therapy was specifically aimed at treating their BD, and 50 (37.6%) reported that the therapy had another focus.

### Concordance with treatment guideline

Concordance with the guideline for each treatment modality was as follows: participation of a psychiatrist in 757 of 773 respondents (97.9%), maintenance pharmacotherapy in 754 of 786 (95.9%), supportive treatment in 560 of 762 (73.5%), use of an emergency plan in 556 of 787 (70.6%), psychotherapy in 399 of 765 (52.2%), group psychoeducation in 371 of 786 (47.2%), and mood monitoring in 369 of 785 respondents (47%). A guideline recommendation for maintenance pharmacotherapy applied to almost all respondents. In only two respondents, both BD I, maintenance pharmacotherapy was not recommended according to the guideline, however one was symptomatic and therefore concordance was scored according to clinical profile “currently symptomatic” as described in Table 1. For 30 respondents, data necessary to determine the need for maintenance pharmacotherapy were either missing or inconclusive.

#### Factors associated with concordance

Factors associated with concordance are presented in Table 3. Specialization for mood disorder of treatment setting, whether the respondents reported that a treatment plan had been made, total years of education, bipolar diagnosis, whether one or more significant others had been asked to participate in the treatment, duration of illness, absence of psychiatric comorbidity, and age were all significantly associated with being better concordant with the guideline. Age and duration of illness were negatively correlated with concordance (see Table 3).

**Table 3:** Demographic, illness related and treatment related factors for concordance with the Dutch guideline for BD: univariate and multivariate analyses

	Univariate model^a^			Multivariate model^b^		
	*B*	*SE*	*P*	*B*	*SE*	*P*
Demographic factors						
Gender: female (male)^c^	− 0.65	1.38	.640			
Age	− 0.15	0.06	.010	0.03	0.07	.670
Marital status: living together or married (living alone, divorced, widowed)^c^	1.34	1.32	.309			
Education: total years of education	0.52	0.15	< .001	0.49	0.16	.002
Illness related factors						
Diagnosis: BD I (BD II, BD NOS or SZA)^c^	3.74	1.34	.005	2.98	1.43	.037
Psychiatric comorbidity: absent (present)^c^	2.97	1.40	.035	2.63	1.50	.080
Duration of illness	− 0.15	0.06	.007	− 0.09	0.07	.193
Treatment related factors						
Treatment setting: specialized centers (non-specialized center)^c^	7.36	1.50	< .001	5.67	1.64	.001
Patient reports that a treatment plan has been made: yes (no)^c^	5.65	1.32	< .001	5.37	1.44	< .001
Significant others have been asked to participate in the treatment: yes (no)^c^	4.86	1.69	.004	3.43	1.81	.059
Patient is involved in decision-making: (never)^c^						
Sometimes	− 0.08	3.09	.979			
Mostly	0.04	2.96	.989			
Always	0.67	2.92	.818			
Other factor						
Patient uses the internet for information on bipolar disorder and treatments: yes (no)^c^	1.56	1.30	.229			

^a^Univariate analysis from simple regression. *Note*: constants in the simple regression models with categorical factors: gender: 72.8, marital status: 72.6, diagnosis: 72.1, psychiatric comorbidity: 72.1, treatment setting: 70.7, whether or not a treatment plan has been made: 72.0, whether or not significant others have been asked to participate: 71.1, patients’ involvement in decision-making: 72.6, and patients’ use of internet:  72.8

^b^The multivariate analysis includes all factors that are univariately associated with concordance (at the level of statistical significance of *α *= 0.05). The coefficient of determination of the multivariate model *R*^2^ = 0.09 (*p* < 0.001)

^c^Reference category

In a multiple regression analysis age, absence of psychiatric comorbidity, duration of illness, and whether or not it was asked to involve significant others in the treatment, did not contribute significantly to the model, although the latter almost reached significance. All other factors were significant. The model explained almost 10% of variance in concordance scores (see Table 3).

## Discussion

In this nationwide study of guideline concordance in routine clinical practice, we found that the use of maintenance pharmacotherapy was highly concordant with the recommendations in the Dutch guideline for BD. This resembles outcomes in some of the previous studies in euthymic or unspecified BD. The high frequency of lithium use (70.6%) in our study is remarkable. In a recent study in Denmark, lithium was prescribed less frequently, 41.7% during a 12-year study period, and its use had declined over the years (Kessing et al. 2016). In contrast to pharmacotherapy, applying psychosocial treatments was much less concordant with the Dutch guideline, even in specialized centers for mood disorders. Especially the low rate of concordance with the participation in (group) psychoeducation is relevant since its efficacy in the maintenance treatment of bipolar disorder has been well established (Colom et al. 2003) and it is thus recommended in the guideline for all BD patients. Moreover, group psychoeducation is widely available in the Netherlands. Concordance-rates for mood monitoring and psychotherapy were also relatively low. For psychotherapy, this may be due to the fact that in the 2008 guideline the indications are still described in general terms. As a consequence, measuring its concordance is less straightforward. Moreover, psychotherapy may have a wider focus than only BD, as was indicated by a considerable number of participants. Specialization of treatment center, years of education, type of diagnosis, and the fact that the patient was informed that a written treatment plan had been made, were all significantly associated with guideline concordance. This is an important finding since some of these factors (making a treatment plan and informing patients about this, and taking into account the level of education of patient) can be easily optimized in everyday clinical practice. Especially the level of understanding of verbal communication can easily be overestimated. Together with inviting a significant other to be involved in the treatment, these findings point in the direction that shared decision-making may result in more guideline-concordant treatments. However, little is currently known if and how shared decision-making may influence clinical outcome in mental health care (Duncan et al. 2010). Although significant in univariate analysis, the presence or absence of psychiatric comorbidity, duration of illness, and age did not contribute significantly in the regression model. In contrast to our hypothesis, gender, marital status, and use of internet by the patient, were not associated with better concordance with the guideline. The involvement of the patient in decision-making in the treatment was also not associated with better concordance, although this was probably due to the fact that the majority of patients stated that they were involved.

### Strengths and limitations

Our study has several strong points. To the best of our knowledge this is a first nation-wide study that it includes a large number of patients in long-term psychiatric outpatient treatment. Moreover, concordance with the treatment guideline was assessed for a wide variety of guideline recommended treatment modalities, and quantified in a composite score taking into account different clinical profiles of patients in maintenance treatment. Dennehey et al. (2005) used a composite score for adherence to the medication guidelines from the Texas Medication Algorithm Project (TMAP). This score measured visit schedules, medication/dosing, and response to patient symptoms. In that study a multifaceted treatment program including the medication guidelines was studied in several intervention clinics, and compared with treatment as usual in non-intervention clinics. Adherence to the guideline was only studied in the intervention clinics. Kilbourne et al. (2010) implemented composite quality metrics to measure the quality of processes of care from various treatment guidelines in a study using medical records, including assessments of symptoms, comorbidity, cardiometabolic outcomes, and documentation of patient treatment experience.

Our study has several limitations. First, although a great effort was made to include a representative cohort in a wide variety of mental health care treatment settings, it is likely that a bias towards psychiatrists and patients with particular interest in our study will have occurred, since many participating patients were treated in specialized mood disorder centers. This will limit the generalizability of our results to non-specialized centers and private practice. Therefore, outcomes may reflect more guideline-concordant care than in settings where psychiatrists (and therefore their patients) did not participate in this study. On the other hand, this could suggest that measures to improve guideline concordance may be even more vital in those settings. A second limitation is that maintenance pharmacotherapy was not assessed in detail but was defined as the use of at least one maintenance drug recommended in the guideline. Whether the choice or dosage of medication was optimized according to guideline recommendations, was not taken into account. Use of antidepressants may have been underreported since information was provided at the initiative of the respondent. As will be the case in studies using patient surveys, not all nuances of individual treatments could be included in the assessment of concordance with the guideline, and answers may have been incomplete or inconsistent. Although an updated guideline was published in 2015, after the study was completed, we assume that the findings of our study are still relevant today, since there were no major changes in the recommendations for long-term treatment strategies in the 2015 guideline. The differences between these guidelines concern recommendations in pharmacotherapy and psychological treatment of a more detailed level than addressed in our study.

## Conclusions

Overall, we conclude that in everyday clinical practice, more than pharmacotherapy, the implementation of psychosocial treatments still needs considerable effort. Actively involving the patient in the treatment may improve concordance rates, although further research in this field is needed. Future studies on psychosocial treatments for bipolar disorder may result in more specific indications for which treatment is needed for which patient in which phase of the illness. Eventually, one can expect that a more personalized approach in treatment guidelines will greatly enhance their utility and implementation.

## Abbreviations

BD: bipolar disorder
SZA: schizoaffective disorder, bipolar type
BD I: bipolar I disorder
BD II: bipolar II disorder
BD NOS: bipolar disorder NOS
